# On the Origin and Evolution of Sperm Cells

**DOI:** 10.3390/cells12010159

**Published:** 2022-12-30

**Authors:** Heidi S. Fisher, Eduardo R. S. Roldan, Tomer Avidor-Reiss, Melissah Rowe

**Affiliations:** 1 Department of Biology, University of Maryland, 1200 Biology-Psychology Building, 4094 Campus Drive, College Park, MD 20742, USA; 2 Department of Biodiversity and Evolutionary Biology, National Museum of Natural Sciences (CSIC), c/Jose Gutierrez Abascal 2, 28006 Madrid, Spain; 3 Department of Biological Sciences, University of Toledo, Toledo, OH 43607, USA; 4 Department of Urology, College of Medicine and Life Sciences, University of Toledo, Toledo, OH 43607, USA; 5 Department of Animal Ecology, Netherlands Institute of Ecology (NIOO-KNAW), 6700 AB Wageningen, The Netherlands

Sperm cells have intrigued biologists since they were first observed nearly 350 years ago by Antonie van Leeuwenhoek and Johan Ham. The discovery of these ‘animalcules’ (i.e., spermatozoa) launched the field of sperm biology, and the subsequent three and half centuries of inquiry into this small, highly specialized cell has revolutionized our understanding of reproduction and fertilization [1]. An extraordinary diversity of sperm morphology and behavior has been observed across the animal kingdom, and the complexity of the molecular and physiological processes that occur as sperm develop, mature, and then eventually undergo their extraordinary journey to find and fertilize an egg continue to intrigue and inspire biologists to this day. At the very heart of the wonderful world of sperm biology lie two central questions: “Why are sperm so small?” and “Why do males produce so many of them?”. Approximately 50 years ago, Geoff A. Parker published two landmark papers that provided evolutionary theory to answer these longstanding questions, theoretical cornerstones upon which much of modern-day evolutionary sperm research are based on. 

In 1970, the publication of “Sperm competition and its evolutionary consequences in the insects” [2] resulted in a paradigm shift in our understanding of sexual selection. Up until this point, sexual selection was viewed as an evolutionary process related to mate acquisition, with sexual selection related to a search for traits that was solely concerned with increasing an individual’s mating success (as originally proposed by Darwin [3]). Darwinian sexual selection thus acts prior to copulation through variations in (typically male) mating success. Although even Darwin himself seemed aware of female multiple mating ([4] and references within), and sperm competition had been observed in Poeciliid fishes [5], it was Parker’s work on the yellow dung fly (*Scathophaga stercoraria*) that catalyzed the field and established the idea that sexual selection continues after insemination (i.e., post-copulatory sexual selection). Parker showed that when females mate with multiple males within a single reproductive period, the sperm of rival males compete to fertilize ova (the post-copulatory equivalent of Darwinian male–male competition). Furthermore, Parker’s work provided new insight into the evolution of associated reproductive traits, such as mating plugs, prolonged copulation, and passive phases, suggesting that such traits may confer a selective advantage when males continue to compete for fertilization success after mating has occurred. Importantly, the insights provided by Parker’s 1970 paper kickstarted a scientific revolution that has subsequently developed into a rich and productive research field. Today, studies of sperm competition number at approximately 250 publications per year [6], and the importance and influence of Parker’s 1970 publication is recognized through numerous reviews (e.g., [4,6,7,8,9,10,11]), as well as a virtual collection of papers published in *Behavioral Ecology* [12] and a theme issue in *Philosophical Transactions of the Royal Society B* celebrating “Fifty years of sperm competition” [13].

In 1972, Parker published another innovative paper in collaboration with RR Baker and VGF Smith—"The origin and evolution of gamete dimorphism and the male–female phenomenon” [14]. This paper provided the first explanation for the evolution of anisogamy and the two sexes that was consistent with the modern evolutionary theory of individual selection, instead of the group selection used in the past. Parker and colleagues’ 1972 paper, and the study of anisogamy more generally, received less attention relative to the 1970 paper on sperm competition (see Figure 3 in [15]); however, this bias may be largely driven by the ease of empirical study of sperm competition. Nonetheless, their 1972 publication was equally groundbreaking and spearheaded revolutionary thinking on one of the central mysteries of evolutionary biology—why do separate male and female gametes exist? Furthermore, these two papers are intricately linked: A major consequence of gamete dimorphism is sperm competition, where the smaller gamete competes for the larger gamete, while sperm competition (or the precursor of sperm competition) drives the evolution of gamete dimorphism. This link is highlighted by Parker himself in this Special Issue, where he writes [15]: “*The paper on the evolution of anisogamy (or gamete dimorphism, as we called it) was also linked to dung flies and sperm competition: thinking about ejaculates competing posed the question of why male and female gametes*…”

Combined, Parker’s two pivotal articles have had a major impact on our understanding of the origin and evolution of sperm cells. The study of post-ejaculatory sexual selection now encompasses a range of sub-fields, including the study of sperm competition, cryptic female choice, and sexual conflict. Furthermore, Parker’s work has contributed to the foundation and development of behavioral ecology as a discipline and inspired much of our own work and that of our colleagues. Now, as both landmark papers pass their 50th year since publication, we aim to celebrate their influence. Thus, the goal for this Special Issue is twofold: to provide a forum for the publication of current research in the field and spark new ideas; and to honor the pioneering work of Parker and serve as a tribute to his original thinking and his many contributions.

This Special Issue contains 22 publications, including original empirical research articles, reviews, and theoretical contributions, and brings together the insights and expertise of more than 80 authors from 14 countries. We are honored that the issue starts with a contribution from Parker himself [15]. Many excellent accounts of Parker’s contributions to the fields of sperm biology, behavioral ecology, and individual selection already exist (e.g., [1,6], as do accounts of Parker’s personal life [16,17]); however, for this Special Issue, Parker provides a personal account of his thinking during the development of his two landmark papers, as well as describing the intellectual environment that formed the backdrop (and sometimes the battlefield) to the development of his ideas. Parker outlines how he began formulating his ideas of sperm competition and gamete dimorphism during his years as an undergraduate and PhD student at Bristol University (Figure 1) working on the dung fly system. We are then taken on a journey through the writing and publication process of both papers before Parker discusses the subsequent developments in the two research fields. Finally, we even catch a glimpse into the doubts that Parker himself had about the usefulness and novelty of his work. Half a century later, there is no doubt about the importance of these contributions, and we thank Parker for sharing his personal account of these papers with us and our many wonderful colleagues in the fields of sperm competition and anisogamy.

Conceptually, the remaining 21 contributions of the Special Issue can be assigned to two broad themes connected to Parker’s two groundbreaking papers. The first theme concerns the origin of sperm cells, or more correctly, the evolution of gametes of two different sizes (i.e., anisogamy), which was the focus of Parker et al.’s 1972 paper [14]. The second theme explores the evolution of sperm and, more generally, post-mating reproductive strategies related to the competitive fertilization success (i.e., sperm competition) following Parker’s 1970 paper [2]. Because the fields of anisogamy and sperm competition have developed unevenly, the papers within this issue reflect this asymmetry; yet, they span an astounding breadth of topics and ideas, similar to the extensive influence Parker has had on the field.

Despite being nearly ubiquitous in nature, the phenomenon of anisogamy has puzzled evolutionary biologists for decades—why are two, and only two, gamete sizes the most evolutionarily stable? Parker and colleagues [14] laid the groundwork for exploring the idea that disruptive selection drove the evolution of large “female” gametes focused on provisioning the resultant embryo, and small “male” gametes focused on search and fusion. Using a theoretical approach, Constable and Kokko [18] relax the assumption that sexual reproduction is obligate (i.e., that gametes can only participate in syngamy or die) to find that parthenogenesis can stabilize isogamy and identify scenarios where parthenogens have an inherent survival advantage. Similarly, Lehtonen [19] derives novel analytical solutions using a game theory approach and offers a theoretical update of Parker et al.’s 1972 model. Combining new results with those from earlier papers, Lehtonen finds that the model is robust to variation in its central components. Taken together, these two studies indicate that the evolution of anisogamy can differ from classically considered trajectories and robustly incorporate real-life features of sexual reproduction.

Following Parker’s 1970 publication, sperm biologists began to consider how sperm competition may drive the evolution of sperm cell morphology [20], a line of inquiry that is ongoing and continues to add new insight into sperm evolution to this day. Three articles in this Special Issue continue along this theme. First, using an experimental evolution approach, Simmons and Garcia-Gonzalez [21] show that selection imposed via sperm competition drives the evolution of sperm cell structure in the dung beetle (*Onthophagus taurus*). Specifically, the authors find that males that evolve under sexual selection produce sperm with longer nuclei compared with those evolving under monogamy, whereas no differences were observed in the lengths of the acrosome or flagella, or the total sperm length [21]. Next, two complementary articles turn to wild populations to determine the adaptive significance of ejaculate traits. First, Kahrl and colleagues [22] show that selection acts on sperm numbers, but not on sperm morphology or velocity, in a wild population of brown anole lizards (*Anolis sagrei*). Similarly, Rowe and colleagues [23] show that sperm numbers are important for within-pair paternity success in red-backed fairy-wrens (*Malurus melanocephalus*), suggesting that sperm numbers serve as a paternity guard under conditions of intense sperm competition. These two articles contribute to a large body of evidence suggesting that selection for large numbers of sperm is a common evolutionary response to sperm competition [24]; however, they also provide examples of differing ejaculate allocation strategies. Whereas male brown anoles are likely producing many small ejaculates and mate frequently, male red-backed fairywrens are suggested to allocate large numbers of sperm to each ejaculate and mate relatively infrequently [22,23].

An increasingly vast body of research now shows that sperm morphology [24] and functional post-ejaculatory sperm modifications [25] are shaped by sperm competition. However, the bulk of this work is focused on aspects of sperm cell size, shape, and physiology, while little attention is paid to the evolution of internal sperm structures. Turner and colleagues [26] address this knowledge gap and examine the association between sperm centriolar composition and fertilization mode (i.e., internal vs. external fertilization strategies) across 277 fish species. They find that internal fertilizers are more likely to produce sperm with atypical centriole composition (i.e., a single centriole vs. the typical two centrioles) or structure [26], which they suggest is associated with a more heterogeneous and challenging environment for sperm to maneuverer within the female reproductive tract. This paper therefore adds to our understanding of how fertilization mode can shape sperm evolution (see also [27,28]).

Key to our understanding of how selection acts on sperm cell morphology is the study of how sperm structure relates to function. Cell structure is highly correlated with function across all taxa and cell types, and the structure–function relationship has been extensively explored for the sperm cell. For example, many theoretical studies predict that sperm length positively correlates with swimming performance, which is supported by early empirical studies, particularly those focused on mammalian species [29,30,31,32]. However, the recent literature highlights the diversity in sperm structure–velocity relationships, with both inter- and intra-specific studies showing positive, negative, and no relationships between sperm morphology and sperm swimming (reviewed in [33,34]). Thus, knowledge of how sperm cell structure translates into performance deserves additional research. Here, Cramer and colleagues [35] show that although sperm from five populations of Canary Islands chiffchaff (*Phylloscopus canariensis*) differ in swimming velocity, this trend is not due to morphological variation among the populations. Moreover, across all populations, the authors find that relatively longer sperm and those with a longer midpiece swim more slowly [35]. This study suggests that sperm movement is more complex than a simple cell length–velocity relationship. This complexity is further highlighted by the work of Hook and colleagues [36], which explores how sperm head shape can influence the cell’s migration and finds that sperm motility is influenced by the apical hook—a hook structure on the head of sperm cells that is a common feature of rodent sperm and has been hypothesized to facilitate sperm–sperm interactions. The authors compare closely related species of *Peromyscus* mice, where sperm aggregation is common, that vary in hook size to show that larger hooks are associated with reduced sperm motility and reduced aggregation size [36].

Understanding how sperm morphology influences sperm motility demands an understanding of the cellular processes underlying the structure–function relationship. Sperm bioenergetics may provide a functional link between sperm morphology and cell performance because energy is crucial for motility, as well as for cell homeostasis and intracellular signaling. In their contribution, Sansegundo and colleagues [37] show that mouse sperm ATP levels decrease over time during capacitation but that there are differences between species regarding ATP consumption and how this impacts on swimming patterns. In mammals, capacitated sperm exhibit hyperactivated motility, which is necessary to achieve fertilization but is energetically costly. These findings suggest that differential ATP consumption during sperm hyperactivation may be a physiological adaptation to increase competition for fertilization success [37]. The significance of sperm hyperactivation is further examined by Hwang and colleagues [38], who show that CatSper proteins, which modulate the influx of Ca^2+^ and regulate hyperactivity, have rapidly evolved in rodents but slowly in a marsupial, the Tasmanian devil (*Sarcophilus harrisii*). Furthermore, the authors show that CatSper sequence homology is high in distantly related marsupial species despite long divergence times (82 MYA) and demonstrate, for the first time, hyperactivated motility via activation of CatSper channels during capacitation in marsupial sperm [38]. Based on their findings, the authors suggest that the molecular characteristics and physiological function of CatSper are evolutionarily conserved within the therian mammals, while possibly being divergent in non-therians.

Taking a different approach to understanding sperm structure-–function relationships, Soulsbury and Humphries [39] provide a biophysical framework for testing mechanistic predictions of how sperm size relates to sperm velocity. The authors use published data from 141 animal species to show empirical support for their “constant speed” model, which predicts that power output is determined by the flagellum and, hence, is proportional to flagellum length [39]. Importantly, they stress that understanding the microenvironment in which sperm must operate is key to understanding the sperm structure–function relationship.

The environment in which sperm operate is, in fact, the focus of four contributions to this Special Issue. This topic is first explored for internally fertilizing species, in which sperm must traverse the complex environment of the female reproductive tract to find and fertilize an egg. The mammalian female reproductive tract, for example, has evolved to achieve two opposing functions: on the one hand it is laden with features that hinder the invasion of pathogens and microorganisms, while on the other hand it must direct sperm to oocytes, a relatively minuscule target at the distal end of a long, complex conduit. In their contribution, Tung and Suarez [40] review how sperm have evolved to move upstream of flow by pushing fluid behind them and accumulating along the walls of the reproductive tract. In addition to these physical interactions, Grewal and colleagues [41] identify genes that regulate interactions between sperm and the female reproductive tract. Using knockdown studies, they show that the *Mst89B* and *CG31287* genes regulate egg fertilization and sperm storage in the female storage organs, without hindering sperm production or transfer [41].

Next, the environment in which sperm operate is explored for externally fertilizing species. First, Green and colleagues [42] describe how sperm from populations of the round goby (*Neogobius melanostomus*) are able to tolerate variable salinity conditions that match their habitat. Sperm of ancestral ecotypes are well adapted to their habitats; yet, contemporary populations shaped by anthropogenic activity also show evidence of sperm adaptation to a given salinity [42]. Devigili and colleagues [43] then explore how sperm locate ova in the externally fertilizing zebrafish (*Danio rerio*). The authors show that sperm accumulate near female reproductive fluid, suggesting that sperm chemoattract toward female fluid to find the ova. Interestingly, this response observed in zebrafish sperm was independent of sperm “quality” traits, including sperm swimming velocity, trajectory, motility, and longevity, suggesting that localization is another trait that may help sperm compete for fertilization success [43]. To complete their study, the authors create a 3D-printed device called a “sperm selection chamber”, reminiscent of a mate choice assay used in behavioral studies, which they have made freely available to the community.

In the 1990s, Parker made further major advances to our understanding of sexual selection with the development of a series of theoretical models examining male ejaculate expenditure strategies under conditions of sperm competition (e.g., [44,45,46,47,48]). Some of these sperm competition games examine the allocation of resources between pre- and post-copulatory episodes of sexual selection [49,50]. These models assume a fixed total budget for total reproductive expenditure, such that there is a trade-off between its two components: expenditure for gaining matings (pre-copulatory competition) and ejaculate expenditure (post-copulatory competition); although, removing the assumption of fixed resource levels and allowing total investment into reproduction to vary can modify the co-variance between pre- and post-copulatory trait expression [51]. Two papers in this Special Issue examine the relationship between pre- and post-copulatory sexual selection. First, Reuland and colleagues [52] assess the evolutionary rates of both pre-and post-copulatory traits, reporting that weapons evolve faster than sperm dimensions. In addition, they find that different components of sperm cells show different evolutionary rates, with the sperm head and midpiece evolving faster than flagellum length [52]. Next, Cramer [53] used a simulation approach to examine the interaction between pre- and post-copulatory sexual selection, exploring whether the two episodes of selection interact synergistically or in opposition. These simulations show that selection on male and sperm traits were independent of one another in within-species contexts, while among-species selection on traits was positively correlated [53], leading Cramer to recommend statistical approaches when examining sperm traits selection in socially monogamous species with extra-pair paternity.

Theoretical sperm competition games also underpin our understanding of how males allocate resources among multiple ejaculate traits [44,45,49]. Studies comparing alternative male reproductive types (e.g., sneaker vs. guarding males) that differ in the risk or intensity of sperm competition have yielded significant insights into our understanding of how sperm competition has shaped ejaculates. Yet, how sperm competition shapes sperm traits in systems with alternative male types remains unclear. In their contribution to the Special Issue, Alonso and colleagues [54] ask how sperm production and sperm traits differ among alternative male types in the ocellated wrasse (*Symphodus ocellatus*), a species with both competition and cooperation among males. The authors find that the male types differ in testes size, ejaculate production, and sperm morphology, but that these patterns differ from those expected based on their behaviorally cooperative roles or predictions based solely on differences among male types in sperm competition. Alonso and colleagues [54] therefore suggest that both reproductive competition and cooperation among males, as well as the potential synergy between the two, can shape sperm traits and ejaculate production in the oscellated wrasse. Thus, this work highlights the importance of considering how individual experience and social interactions among males can influence patterns of sperm production and characteristics.

Importantly, trade-offs are also expected between competing life history functions and traits, such as future reproduction and survival [55]. By experimentally increasing ejaculate production rates, McMahon and colleagues [56] explore potential trade-offs between ejaculate expenditure and (i) somatic maintenance, (ii) future reproduction, and (iii) male longevity and long-term survival. Furthermore, because a limited energy budget is central to trade-offs, McMahon and colleagues [56] maintained males on either a high or low feeding regime. Although the authors found no evidence for reproductive trade-offs, they show that resource limitation imposes significant costs on males, i.e., males maintained under food limitation show reduced body mass, lower sperm viability, and decreased survival. These findings suggest that while the costs of spermatophore production may be low, they also point towards the condition dependence of sperm viability [56].

The realization that there are considerable differences in sperm numbers and morphology across species, and even across males within species, begs the question of how sperm develop within the testis, an aspect of evolutionary developmental biology (the field of “evo-devo”). By bringing together molecular genetics with developmental patterns, as well as phylogenetics and evolutionary theory, the paper by Syed and colleagues [57] is a valuable step in this direction. Taking advantage of the highly diverse *Drosophila* model, the authors report on the discovery of a biological novelty—sperm cyst looping—that enables males to produce extremely long sperm in relatively short testis. The authors show that sperm cyst looping is present in members of two related species groups (the *willistoni* and *saltans* groups, with complete and partial looping, respectively), and demonstrate a pattern of co-diversification between sperm length and the length of the female’s seminal receptacle [57]. Furthermore, the authors suggest that by removing the allometric constraint on sperm length, sperm cyst looping appears to allow males to evolve extremely long sperm for their body mass while also avoiding delays in reproductive maturation. They conclude their work by speculating on the evolutionary origin and maintenance of this unique adaptation, setting the stage for a new line of research that could provide novel insights into sperm evolution.

Finally, cutting across the two themes of this Special Issue, Van Goor and colleagues [58] explore the evolution and maintenance of sex ratios in nematodes, a diverse metazoan phyla exhibiting remarkable variation in reproductive mode and sex ratio. In their review, the authors provide evidence that several mechanisms likely underlie the highly female-biased sex ratios observed in some nematode species, including self-fertile hermaphroditism or facultative parthenogenesis, non-Mendelian meiotic oddities involving the sex chromosomes, and environmental sex determination. Intriguingly, the elimination or scarcity of males can have consequences for a range of reproductive processes, including, among other things, reducing the strength of sexual selection and post-mating pre-zygotic reproductive isolation [58]. 

Together, the articles in this Special Issue demonstrate the enormous breadth of current and ongoing research into this tiny haploid cell, as well as the extraordinary impact that Parker has had on the field of sperm biology. The clear evolutionary thinking laid out in Parker’s many contributions resulted in well-defined, testable hypotheses of sperm evolution, to which a curious and dedicated community of sperm biologists have pursued answers. This has resulted in massive leaps in our understanding of, among other things, sperm form and function, reproductive strategies, sperm development, and how the environments in which sperm operate shape sperm evolution. However, the contributions to this Special Issue also highlight how much we still do not know. We hope that this issue both adds to our knowledge base and stimulates new studies. Whether it be studies of the variations in the reproductive modes of nematodes, examining the interaction between competition and cooperation in fish, or investigating how the female reproductive tract influences sperm evolution, we are certain that the pioneering work of Geoff A. Parker will continue to inspire our science, and that of our colleagues, for years to come.

## Figures and Tables

**Figure 1:**
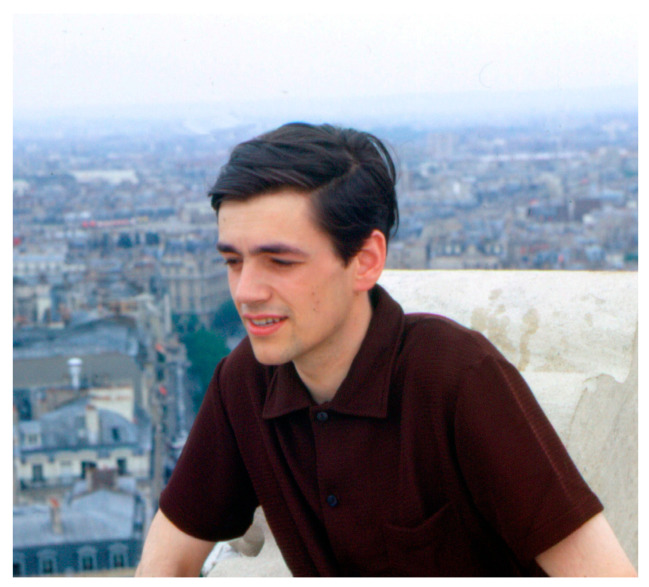
Geoff A Parker in 1967 during his PhD at University of Bristol.
